# In-depth characterization of a novel live-attenuated Mayaro virus vaccine candidate using an immunocompetent mouse model of Mayaro disease

**DOI:** 10.1038/s41598-020-62084-x

**Published:** 2020-03-24

**Authors:** Mânlio Tasso de Oliveira Mota, Vivian Vasconcelos Costa, Michelle Amantéa Sugimoto, Georgia de Freitas Guimarães, Celso Martins Queiroz-Junior, Thaiane Pinto Moreira, Carla Daiane de Sousa, Franciele Martins Santos, Victoria Fulgêncio Queiroz, Ingredy Passos, Josy Hubner, Danielle Gloria Souza, Scott C. Weaver, Mauro Martins Teixeira, Maurício Lacerda Nogueira

**Affiliations:** 1 Faculdade de Medicina de São José do Rio Preto, São José do Rio Preto, São Paulo, Brazil; 2 Institute of Biological Sciences, Universidade Federal de Minas Gerais, Belo Horizonte Minas Gerais, Brazil; 3 World Reference Center for Emerging Viruses and Arboviruses, Institute for Human Infections and Immunity, and Department of Microbiology and Immunology, University of Texas Medical Branch, Galveston, Texas 77555-0610 USA

**Keywords:** Live attenuated vaccines, Viral infection

## Abstract

Mayaro virus (MAYV) is endemic in South American countries where it is responsible for sporadic outbreaks of acute febrile illness. The hallmark of MAYV infection is a highly debilitating and chronic arthralgia. Although MAYV emergence is a potential threat, there are no specific therapies or licensed vaccine. In this study, we developed a murine model of MAYV infection that emulates many of the most relevant clinical features of the infection in humans and tested a live-attenuated MAYV vaccine candidate (MAYV/IRES). Intraplantar inoculation of a WT strain of MAYV into immunocompetent mice induced persistent hypernociception, transient viral replication in target organs, systemic production of inflammatory cytokines, chemokines and specific humoral IgM and IgG responses. Inoculation of MAYV/IRES in BALB/c mice induced strong specific cellular and humoral responses. Moreover, MAYV/IRES vaccination of immunocompetent and interferon receptor-defective mice resulted in protection from disease induced by the virulent wt MAYV strain. Thus, this study describes a novel model of MAYV infection in immunocompetent mice and highlights the potential role of a live-attenuated MAYV vaccine candidate in host’s protection from disease induced by a virulent MAYV strain.

## Introduction

*Mayaro virus* (MAYV) is an arbovirus of the *Togaviridae* family, *Alphavirus* genus. It is enzootic in South America and endemic in some rural areas^1^. Since its discovery in 1954 at Mayaro county, Trinidad and Tobago^2^, MAYV has been reported in different countries of Latin America and the Caribbean^3,4^. In Brazil the first MAYV outbreak was reported in 1957, near the River Guama, in Pará State^5^. However, most of the clinical and epidemiologic knowledge on this virus came from an outbreak that occurred in Belterra, Pará State, in 1978^6,7^. Since then, MAYV circulation has been reported in several Brazil States, mainly in the Central-West and North regions^8–13^ including Pará, Tocantins, Mato Grosso, Amazonas and Goiás^1,4–10^. Although it was initially restricted to forest areas, mainly in the Amazon region^14^, there is growing evidence of MAYV spread to other regions of Brazil, making it an emerging new threat along with other endemic arboviruses such as dengue, zika and chikungunya viruses.

Canopy-dwelling mosquitoes of the *Haemagogus* genus transmit MAYV. Despite generally being confined to forested areas, it has been shown that there is potential for urbanization and consequently generation of large epidemics. A similar phenomenon was recently reported for chikungunya virus (CHIKV), a genetically and antigenically related arthritogenic alphavirus that acquired mutations that allowed it to be transmitted efficiently by additional anthropophillic *Aedes* mosquitoes^15,16^. It has been demonstrated that MAYV can also be transmitted by *Aedes* mosquitoes^17,18^. Since many people work or live in forested areas and due to the ability of MAYV to be transmitted by *Aedes* mosquitoes, higher number of Mayaro fever (MF) cases could occur in the coming years. For example, there are rising concerns about a broader circulation of this virus to other countries of Central America and Caribe. For example, MAYV was identified in 2010 as the etiologic agent of a febrile illness with arthralgic manifestations that occurred in 77 individuals, in which 19 cases were confirmed as seropositive^19^. Another study conducted by Terzian and colleagues (2015) reported the complete genome sequence of a MAYV isolated from a symptomatic patient in Acre/Brazil^20^. Authors have shown that MAYV belongs to the genotype D which is very close to the Bolivian strains^20^. Finally, a MAYV case in Haiti^19^ was particularly important because this country is a central route of entrance of this arbovirus in the southern regions of the USA, as previously observed for chikungunya and zika virus^21^.

MF is a dengue-like febrile syndrome. The incubation phase lasts for 7 to 12 days, followed by onset of high fever, frontal headache, arthralgia, arthritis, myalgia, articular edema (mainly in wrist, knees and ankles), retro-orbital pain, malaise, skin rash, vomiting, diarrhea among others. Arthralgia and arthritis are common hallmarks of MF, which is painful and very debilitating, sometimes lasting for weeks-to-years after the clearance of infection^22^. The similarity of the clinical presentation of MF with other arboviral diseases, such as dengue, along with the absence of good diagnostic kits in areas of co-circulation of multiple arboviruses suggest that the precise number of cases is probably underestimated and the incidence of MAYV infection could be much higher^10,13,22^, e.g., it has been suggested that about 41.5% of riverside inhabitants have antibodies against MAYV^23^. Another study estimated that about 1% of all dengue-like diseases in northern Latin America could be due to MAYV infections^24^. The previous studies suggest that circulation of MAYV is probably broader and the number of cases higher than currently reported. Nevertheless, there is no approved vaccine or specific treatment for MF and the development of a vaccine is essential to prevent future outbreaks of this potentially important human disease.

Here, using a wild-type (WT) strain of MAYV, we developed a model of MAYV infection in immunocompetent mice that emulates several aspects of the human disease, including arthritis and hypernociception. In parallel, we evaluated the response profile of a live-attenuated MAYV vaccine candidate (MAYV/IRES)^25,26^ by conducting an in-depth characterization of clinical, virological and immunological parameters in comparison to the WT strain. Finally, we investigated the protective role of MAYV/IRES vaccination in both immunocompetent and A129^−/−^ (interferon type I receptor-defective) mice followed by a challenge with the virulent wt MAYV strain.

## Results

### Characterization of MAYV disease in immunocompetent mice

**Figure 1:**
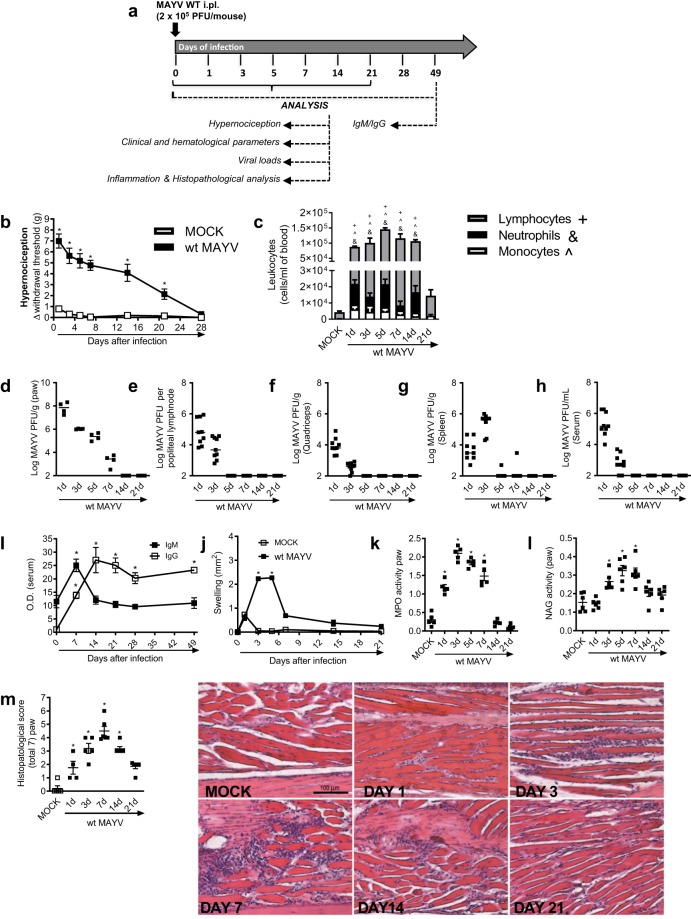
Characterization of MAYV disease in immunocompetent mice. (**a**) Experimental scheme. 6-week-old BALB/c mice were infected or not with wt MAYV (2 × 10^5^ PFU/50 μL, i.pl.) and several analyses were performed along the kinetic of infection. (**b**) Mechanical hypernociception was assessed daily. Results are shown as the differences (Δ) between the force (g) necessary to induce dorsal flexion of the tibio-tarsal joint, followed by paw withdraw, calculated by subtracting zero-time mean measurements before MAYV inoculation from the time interval mean measurements after infection. (**c**) Total and differential cell counts of inflammatory cells in the blood. (**d–h**) Plaque assay analysis of hind paw (**d**), LNP (**e**), quadriceps muscle (**f**), spleen (**g**) and serum (**h**). (**i**) Anti-MAYV IgM and IgG titers of pre- and post-infection serum samples collected on day zero and every 7 days until day 49. (**j**) MAYV-induced footpad swelling was assessed daily by measuring the height and width of the perimetatarsal area of the hind foot. Results are shown as mm^2^. (**k**) Neutrophil influx to the hind foot was measured indirectly by evaluation of MPO activity. (**l**) Macrophage influx to the hind foot was measured indirectly by analysis of NAG activity. (**m**) Shows semi-quantitative analysis (histopathological score) of hind paw sections of mock- and MAYV-infected mice, 1, 3, 7, 14 and 21 d.p.i. Representative pictures from hind paw sections. Results were expressed as median (**d–h**) or mean ± SEM (**b**,**c** and **i–m**) and are representative of two experiments. Original magnification: 200×. Scale bar: 100 µm. *p < 0.05 when compared to control uninfected mice (MOCK), as assessed by two-way (**b**,**i**,**j**) or one-way ANOVA followed by Newman-Keuls post-test (k-m). ^+, &^, ^p < 0.05 (for lymphocytes, neutrophils and monocytes, respectively) when compared to control uninfected mice (MOCK), as assessed by one-way ANOVA followed by Newman-Keuls post-test (**c**).

Experiments were conducted in 6-week-old immunocompetent (BALB/c strain) mice inoculated with 2 × 10^5^ PFU/50 μL of MAYV via the s.c. intraplantar (i.pl.) route and several analyses were performed to assess kinetics of infection (Fig. 1a). Non-infected mice (MOCK) received a 50 μL s.c. i.pl. injection of C6/36 cell culture supernatant. Figure 1b shows that MAYV-infected mice resulted in hypernociception from day 1 until day 21 after virus inoculation and this was much greater than responses observed in control littermates. At day 28, all values returned to baseline levels (Fig. 1b). Total and differential blood leukocyte analysis revealed elevated numbers of monocytes, neutrophils and lymphocytes on the MAYV-infected group, from day 1 to day 14, in comparison to non-infected controls. At day 21, all leukocyte subtypes returned to baseline values (Fig. 1c). Viable viruses were recovered from the paw at days 1 to 7 p.i (Fig. 1d), while in the popliteal lymph node (LNP) (Fig. 1e), quadriceps muscle (Fig. 1f), spleen (Fig. 1g) and serum (Fig. 1h) virus recovery occurred at days 1 and 3 post-infection. Of note, no detectable virus was recovered from the liver at any analyzed time points (data not shown).

Next, we evaluated whether MAYV infection could induce specific anti-MAYV responses. Figure 1i revealed that IgM levels were elevated at day 7 post-MAYV inoculation and then returned to baseline levels (Fig. 1I- black square) while IgG levels started to increase at day 7 reaching a peak at day 14 and remained elevated until day 49 (last time point analyzed) (Fig. 1i – white square).

Plantar edema was observed in MAYV-infected mice at days 3 and 5 p.i (Fig. 1j). Neutrophil influx, as determined by MPO activity, was elevated from day 1 until day 7 after MAYV inoculation (Fig. 1k), while macrophages levels, measured by NAG activity, started to increase at day 3 and remained high until day 7 (Fig. 1l). At days 14 and 21, both MPO and NAG levels, returned to baseline values (Fig. 1k,l). Finally, histopathological analysis revealed that MAYV infection induced a time-dependent inflammatory reaction in the hind paws of mice. From days 1 to 3 post-infection, a discrete PMN infiltrate was observed diffusely within muscle fibers beneath the skin. The number of cells peaked at day 7, when neutrophils partially replaced muscle layer inducing loss of muscle architecture. Superficial bone resorption or synovial alterations in joints were not prominent. The inflammatory scenario declined from day 7 on, when mononuclear cells were seen more frequently. Tissues returned to basal conditions on day 21-post infection (Fig. 1m). No evident histopathological alterations were detected in knee joint samples (Fig. S1). Overall, our results demonstrate that MAYV inoculation into immunocompetent mice results in a self-limited disease similar to MF seen in humans.

### MAYV infection of WT mice is associated with acute production of inflammatory mediators

**Figure 2:**
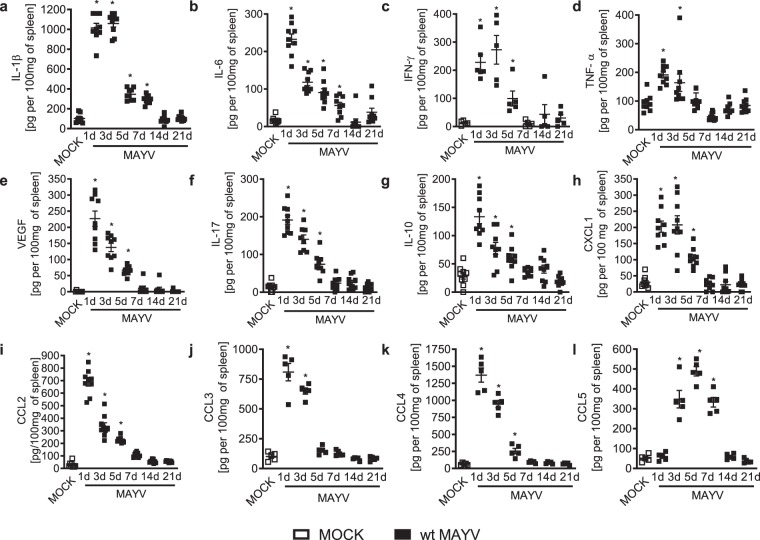
MAYV infection is associated with acute production of inflammatory mediators. 6-week-old BALB/c mice were inoculated or not with wt MAYV (2 × 10^5^ PFU/50 μL, i.pl.) and levels of (**a**) IL1-β; (**b**) IL-6; (**c**) IFN-γ; (**d**) TNF-α; (**e**) VEGF; (**f**) IL-17; (**g**) IL-10; (**h**) CXCL-1; (**i**) CCL2; (**j**) CCL3; (**k**) CCL4 and (**l**) CCL5 in the spleen were analyzed by ELISA in different time-points. Results are expressed as pg/100 mg of spleen. Data is representative of two experiments expressed as mean ± SEM. *p < 0.05 when compared to control uninfected mice (MOCK), as assessed by one-way ANOVA followed by Newman-Keuls post-test.

**Figure 3:**
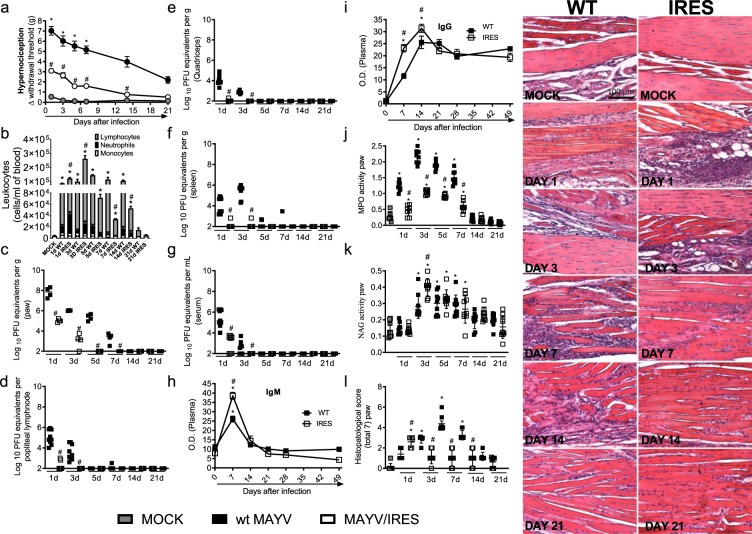
The MAYV/IRES attenuated vaccine strain induces stronger cellular and humoral responses in BALB/c mice. 6-week-old BALB/c mice were inoculated or not with either wt MAYV or MAYV/IRES (2 × 10^5^ PFU/50 μL, i.pl.). (**a**) Mechanical hypernociception was assessed at different time points after virus inoculation, as described in figure legend 1. (**b**) Total and differential cell counts of inflammatory cells in the blood. (**c–g**) plaque assay analysis of hind paw (**c**) LNP (**d**), quadriceps muscle (**e**), spleen (**f**) and serum (**g**). (**h**,**i**) Anti-MAYV IgM and IgG titers of pre- and post-infection serum samples collected on day zero and every 7 days until day 49. (**j**) Neutrophil influx to the hind foot was measured indirectly by evaluation of MPO activity. (**k**) Macrophage influx to the hind foot was measured indirectly by analysis of NAG activity. (**l**) Shows semi-quantitative analysis (histopathological score) and representative pictures of hind paw sections of control and MAYV-infected mice, 1, 3, 7, 14 and 21 d.p.i. Results were expressed as median (**c–g**) or mean ± SEM (**a**,**b** and **h–l**) and are representative of two experiments. Original magnification: 200×. Scale bar: 100 µm. *p < 0.05 when compared to control uninfected mice (MOCK), as assessed by two-way (a, i and h), one-way ANOVA followed by Newman-Keuls post-test (**b–g**, **j**,**k**) or Mann-Whitney test (**l**).

Long-term arthralgia after MAYV infection is associated with sustained pro-inflammatory cytokine responses^27^. We evaluated the production of inflammatory mediators during the course of MAYV infection in BALB/c mice. MAYV infection induced acute production of several pro-inflammatory molecules in the spleen such as IL1-β (Fig. 2a) and IL-6 (Fig. 2b) from days 1 to 7 of infection; IFN-γ (Fig. 2c) from days 1 to 5 and finally, TNF-α (Fig. 2d) at the early time-points of 1 and 3 days post-MAYV inoculation. Indeed, the growth factor VEGF (Fig. 2e), the cytokine IL-17 (Fig. 2f) and the anti-inflammatory cytokine IL-10 (Fig. 2g) were also elevated from days 1 to 5 post-MAYV inoculation. Finally, several chemokines involved in leukocyte recruitment were augmented upon MAYV-inoculation: CXCL-1, involved on the recruitment of neutrophils (Fig. 2h), CCL2 of monocytes (Fig. 3i); CCL3, CCL4 and CCL5 associated with recruitment of monocytes, dendritic cells and activated T cells (Fig. 2j–l). Most of those chemoattractants were elevated from day 1 to day 5 except for CCL3, which was augmented only from days 1 to 3 and CCL5 that increased at day 3 and lasted until day 7. Of note, all mediators returned to baseline levels as in MOCK controls by day 14 (Fig. 2a–l). In serum, elevated levels of IL1-β were detected at days 1 and 3 after MAYV inoculation (Fig. S2a) but were no detectable levels of TNF-α or IL-6 (Fig. S2b,c).

### MAYV/IRES induces reduction in viral loads and better immunecellular response in WT mice

MAYV causes an acute debilitating disease that is associated with long-term arthralgia and persistent pain in more than 50% of the affected individuals^27^. There is no specific treatment or available vaccine to treat MAYV infection. Here, we investigated the effects of the live-attenuated MAYV/IRES vaccine^25,26^ further by using the immunocompetent mouse model described above. Results showed that while mice infected with the wt MAYV strain presented elevated hypernociception from day 1 until day 21 of virus inoculation (Figs. 1b and 3a), the intensity and duration of hypernociception was significantly reduced in the MAYV/IRES-inoculated group (Fig. 3a). By day 14, nociceptive levels in MAYV/IRES mice returned to levels similar to those observed in MOCK-controls (Fig. 3a). Interestingly, an acute increase in circulating neutrophils and lymphocytes (days 1 and 3) was found in MAYV/IRES mice, in comparison to wt MAYV infected controls. At days 7 and 14, values were significantly reduced in comparison to wt MAYV littermates (Fig. 3b). Of note, by day 21, all leukocytes subtypes returned to baseline values similar to those found in MOCK controls (Fig. 3b). As expected, the recovery of infectious virus was greatly less in MAYV/IRES-infected mice, as shown in the paw (Fig. 3c), popliteal lymph node (LNP) (Fig. 3d), quadriceps muscle (Fig. 3e), spleen (Fig. 3f) and serum (Fig. 3g) in comparison to the wt MAYV strain. Specific anti-MAYV IgM and IgG responses were greater in the MAYV/IRES group than in wt MAYV-infected littermates. Figure 3h shows there was a 2-fold greater increase in IgM levels in the MAYV/IRES group, at day 7 after virus inoculation, when compared to WT virus. Similarly, IgG levels were massively augmented at days 7 and 14 post-MAYV/IRES inoculation (Fig. 3i). However, after day 21, IgG levels were equally elevated in both infected groups in comparison to MOCK controls (Fig. 3i).

The specificity of the antibodies elicited in this model was determined by PRNT_50_. Sera from MAYV/IRES-inoculated mice were tested against the wt MAYV strain *in vitro*. Supplementary Table 1 shows that neutralizing antibodies were induced by MAYV/IRES inoculation, reaching satisfactory titers 5 days after inoculation. To determine if this response could cross-react with other alphaviruses, a pool of sera from five animals inoculated with MAYV/IRES strain and collected at each time point was tested against CHIKV (Sup. Table 2). PRNT_50_ values revealed that the MAYV/IRES vaccine is effective in inducing humoral immunity against MAYV but not CHIKV.

Inoculation of MAYV/IRES did not induce any plantar edema (data not shown). Neutrophils but not macrophages levels on mice hind paw, assessed by MPO and NAG activity, respectively, were less in MAYV/IRES mice than in mice infected with the WT virus (Fig. 3j,k). Despite the neutrophil reduction at day 3 after infection, NAG levels were greater in MAYV/IRES mice than in wt MAYV infected mice (Fig. 1k). Finally, histopathological analysis of mice hind limbs revealed greater cellular infiltrated, characterized mostly by neutrophils, on the hind paw of MAYV/IRES at day one after virus inoculation, followed by a massive decrease of this infiltrate in the next time points when compared to wt MAYV-inoculated controls (Fig. 3l). No evident histopathological changes were detected in knee joints of either wt MAYV or MAYV/IRES groups (Fig. S3). Taken together, these results show that the administration of the attenuated MAYV/IRES vaccine was safe to the immunocompetent host, induced significantly less hypernociception and was associated with the induction of stronger local cellular and systemic humoral responses.

### Cytokine and cellular responses to attenuated MAYV/IRES vaccine

**Figure 4:**
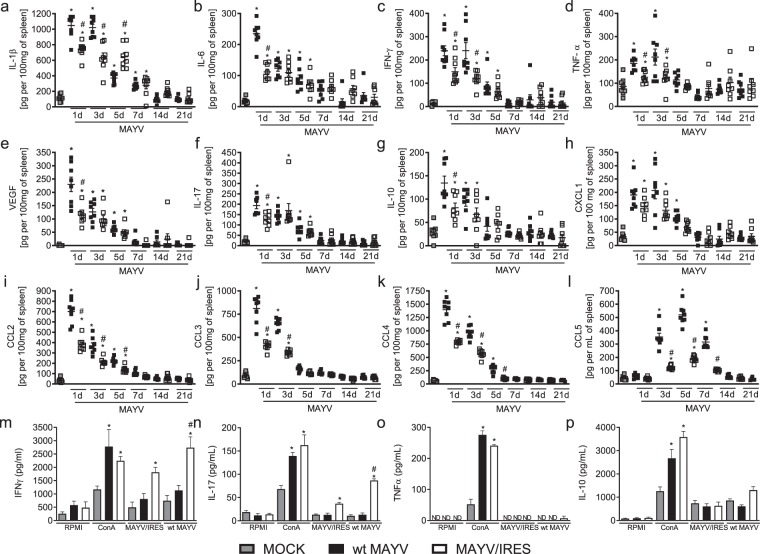
The MAYV/IRES attenuated vaccine strain is associated with reduced production of inflammatory mediators *in vivo*, and upon antigen stimulation *ex vivo*. 6-week-old BALB/c mice were inoculated either wt MAYV or MAYV/IRES (2 × 10^5^ PFU/50 μL, i.pl.) and levels of (**a**) IL1-β; (**b**) IL-6; (**c**) IFN-γ; (**d**) TNF-α; (**e**) VEGF; (**f**) IL-17; (**g**) IL-10; (**h**) CXCL-1; (**i**) CCL2; (**j**) CCL3; (**k**) CCL4 and (**l**) CCL5 in the spleen were measured in different time-points. Results are expressed as pg/100 mg of spleen. (**m–p**) *Ex vivo* stimulation of splenocytes isolated from MOCK-, wt MAYV-, and MAYV/IRES-inoculated mice 28 d.p.i. Splenocytes were then re-stimulated with 2 μg/mL ConA, inactivated wt MAYV or inactivated MAYV/IRES. Cell supernatants were harvested after 48 hours of stimulation for cytokine (**m**) IFN-γ; (**n**) IL-17; (**o**) TNF-α and (**p**) IL-10 measurements as described in methods. Results are expressed as pg/100 mL of culture supernatant. Data is representative of two experiments expressed as mean ± SEM. *p < 0.05 when compared to control uninfected cells (MOCK). ^#^p < 0.05 when compared to wt MAYV stimulated cells, as assessed by one-way ANOVA followed by Newman-Keuls post-test. ND, not detectable.

An ideal MAYV vaccine would produce a strong, rapid and long-lived immunity after a single dose to rapidly control outbreaks, with a low risk of adverse side effects^25^. Infection of BALB/c mice with WT MAYV induced acute production of pro-inflammatory cytokines (Figs. 2a–d,f and 4a–d,f), growth factors (Figs. 2e and 4e), anti-inflammatory cytokines (Figs. 2g and 4g) and several chemokines (Figs. 2h–l and 4h–l). We also quantified the levels of inflammatory mediators after the inoculation of the attenuated MAYV/IRES vaccine. In general, MAYV/IRES inoculation induced milder inflammatory response in comparison to wt MAYV (Fig. 4a–l). More specifically, IL-1β levels were reduced at day 3 post-MAYV/IRES inoculation and augmented at day 5 (Fig. 4a). IL-6 (Fig. 4b), VEGF (Fig. 4e), IL-17 (Fig. 4f) and IL-10 (Fig. 4g) was reduced only at day 1 post-MAYV/IRES inoculation compared to WT MAYV strain while IFN-γ (Fig. 4c) and TNF-α (Fig. 4d) were diminished at days 1 and 3 post-MAYV/IRES inoculation. Finally, no differences were found between infected groups on CXCL-1 levels (Fig. 4h); however, CCL2 (Fig. 4i), CCL3 (Fig. 4j), CCL4 (Fig. 4k) and CCL5 (Fig. 4l) were reduced from day 1 to day 7 in the MAYV/IRES-inoculated group. Therefore, our results demonstrate that, in addition to the increased cellular and humoral responses observed on the live attenuated MAYV/IRES vaccine group, there was an important reduction in levels of systemic inflammatory mediators, suggesting the generation of a more controlled and effective immune response.

Next, we decided to confirm the ability of the MAYV/IRES vaccine to induce cellular responses against MAYV challenge *ex vivo*. Spleens from non-infected and wt MAYV or MAYV/IRES-infected mice were collected, splenocytes isolated and plated. Two hours later, media was harvested and splenocytes stimulated with one of the following: media, Con-A (2 µg/mL), inactivated MAYV/IRES (iMAYV/IRES) or inactivated wt MAYV (iwt MAYV) for 48 hours followed by investigation of cytokine levels in the supernatant. Figure 4m–p shows that splenocytes from the MAYV/IRES vaccine group stimulated with either iMAYV/IRES or iwt MAYV were able to produce IFN-γ (Fig. 4m) and IL-17 cytokines (Fig. 4n) but not IL-10 (Fig. 4o) or TNF-α (Fig. 4p). Interestingly, cytokine production in response to iwt MAYV was higher in comparison to iMAYV/IRES–stimulated splenocytes (Fig. 4m,n). Negative control cells produced low levels of the tested cytokines, while the polyclonal stimuli concanavalin A (ConA, positive control) induced elevated levels of all tested mediators (Fig. 4m–p). These results demonstrate that MAYV/IRES vaccine is able to induce rapid, strong and long lasting cellular and humoral responses against MAYV.

### Vaccination with MAYV/IRES protects mice from wild-type MAYV challenge

**Figure 5:**
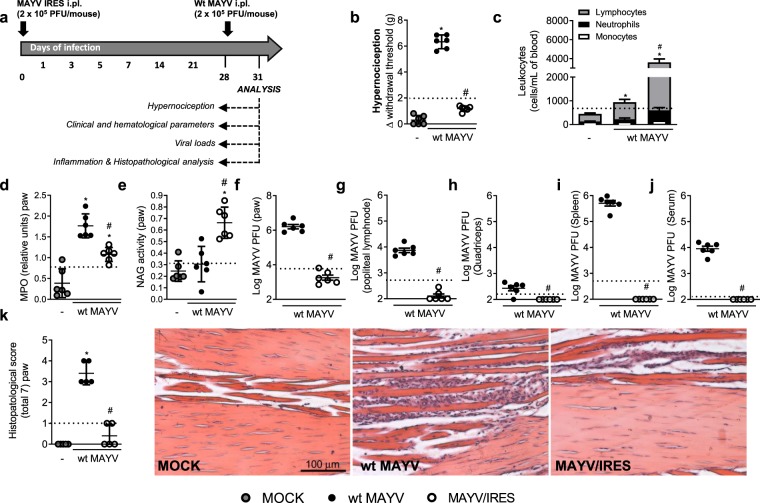
Vaccination with MAYV/IRES strain protects mice from wild-type MAYV challenge. Six-week-old BALB/c mice were inoculated or not with MAYV/IRES (2 × 10^5^ PFU/50 μL, i.pl.) and 28 days later mice were challenged with 2 × 10^5^ PFU/50 μL of WT-MAYV at the same hind paw. Three days later, several analyses were performed. (**a**) Experimental design. (**b**) Mechanical hypernociception was assessed at day 3 after virus inoculation, as described in figure legend 1. (**c**) Differential cell counts on blood were represented as number of leukocytes, mononuclear cells and neutrophils normalized on % of total cells counts. (**d**) Neutrophil influx to the hind foot was measured indirectly by evaluation of MPO activity. (**e**) Macrophage influx to the hind foot was measured indirectly by analysis of NAG activity. (**f–j**) Plaque assay analysis of hind paw (**f**) PLN (**g**), quadriceps muscle (**h**), spleen (**i**) and serum (**j**). Results are shown as the log of PFU per/g of tissue or PFU per/mL of serum. (**k**) Shows semi-quantitative analysis (histopathological score) after Hematoxylin & Eosin staining of hind paw sections of control and MAYV-infected mice three days after wt MAYV inoculation. Representative pictures from hind paw sections. MOCK- not-infected. Results were expressed as median (**f–j**) or mean ± SEM (**a–e** and **k**) and are representative of two experiments. Original magnification: 200×. Scale bar: 100 μm. * for p < 0.05 when compared to control uninfected mice (MOCK). ^#^p < 0.05 when compared to naïve wt MAYV infected group, as assessed by one-way ANOVA followed by Newman-Keuls post-test. Dashed lines are representative of naïve mice, which received MAYV/IRES.

Once we demonstrated that MAYV/IRES inoculation to BALB/c mice resulted in long-lasting cellular and humoral responses, we decided to evaluate if vaccination of BALB/c could eliminate or minimize MAYV-induced disease. For that, 6-week-old BALB/c mice were inoculated with 2 × 10^5^ PFU/50 μL of MAYV/IRES attenuated virus via the s.c. intraplantar (ipl) route and 28 days later, when virus was cleared, and elevated neutralizing IgG levels were detected in serum (Fig. 3i and Sup. Table 1), they were challenged with the wt MAYV strain at same inoculum (2 × 10^5^ PFU/50 μL ipl). Analyses were performed three days after wt MAYV virus inoculation. The experimental design is shown in Fig. 5a. Results showed that vaccination prevented the occurrence of most clinical outcomes associated with MAYV disease (Fig. 5). Specifically, while wt MAYV inoculation to naïve mice induced articular hypernociception, MAYV/IRES vaccinated mice were completed protected (Fig. 5b). As shown in Figs. 3 and 4, MAYV/IRES administration to naïve mice induced a mild increase in articular hypernociception as compared to wt MAYV. As a control for this experiment, the dashed line in Fig. 5 represents naïve mice that only received MAYV/IRES and were analyzed 3 days later. Figure 5c shows that MAYV/IRES vaccination elicited increased cellular responses, characterized by elevation of neutrophils and lymphocytes in the bloodstream. Accordingly, reduced MPO (Fig. 5d) and increased NAG (Fig. 5e) levels on the inoculated hind paw were detected on vaccinated group as compared to wt MAYV–infected naïve mice.

Plaque assay revealed massive reduction of infectious virus recovery from the hind paw (Fig. 5f) and a complete prevention of virus spread to the PLN (Fig. 5g), quadriceps muscle (Fig. 5h), spleen (Fig. 5i) and serum (Fig. 5j).

**Figure 6:**
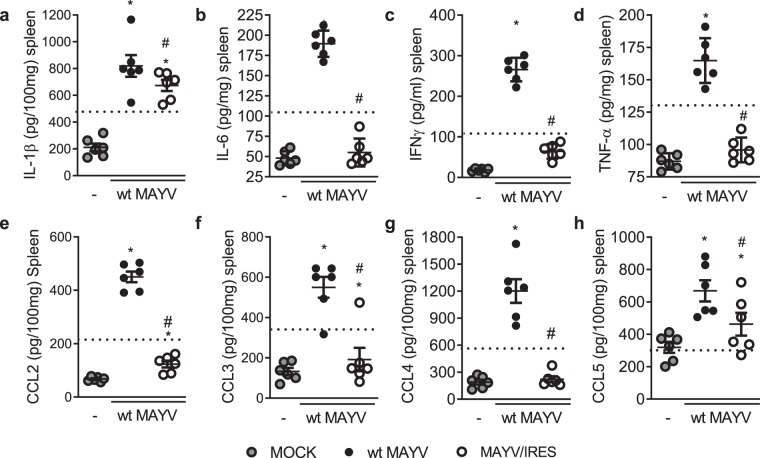
Vaccination with MAYV/IRES strain attenuates production of inflammatory mediators after wt MAYV challenge. Six-week-old BALB/c mice were inoculated or not with MAYV/IRES (2 × 10^5^ PFU/50 μL, i.pl.) and 28 days later mice were challenged with wt MAYV (2 × 10^5^ PFU/50 μL, i.pl.) at the same hind paw. Three days later, protein levels of (**a**) IL1-β; (**b**) IL-6; (**c**) IFN-γ; (**d**) TNF-α; (**e**) CCL2; (**f**) CCL3; (**g**) CCL4 and (**h**) CCL5 were analyzed in the spleen. Results are expressed as pg/100 mg of spleen. Data is representative of two experiments expressed as mean ± SEM. MOCK- not-infected. ^*^for p < 0.05 when compared to control uninfected mice (MOCK). ^#^ for p < 0.05 when compared to naïve wt MAYV infected group, as assessed by one-way ANOVA followed by Newman-Keuls post-test. Dashed lines indicate naïve mice that received MAYV/IRES.

Histopathological analysis from the hind paw of MAYV/IRES-vaccinated mice revealed maintenance of tissue architecture, in contrast to the disruption caused by WT MAYV injection (Fig. 5k). ELISA analysis from the spleen of vaccinated mice revealed a reduction in levels of the cytokines IL-1β (Fig. 6a), IL-6 (Fig. 6b), IFN-γ (Fig. 6c) and TNF-α (Fig. 6d) as well as the chemokines, CCL2 (Fig. 6e), CCL3 (Fig. 6F), CCL4 (Fig. 6g) and CCL5 (Fig. 6h).

**Figure 7:**
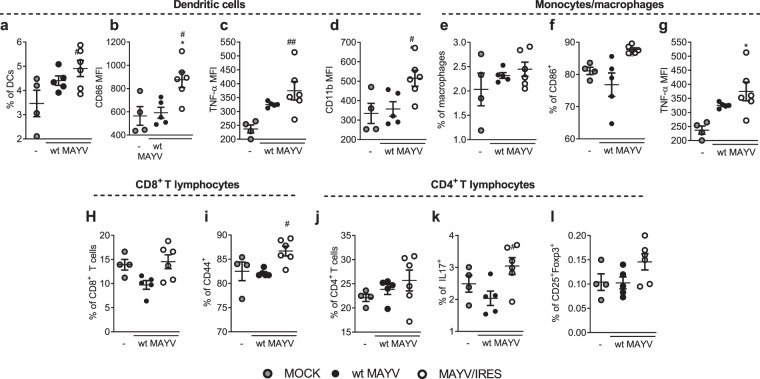
Vaccination with MAYV/IRES strain induces a better splenic immune response after wt MAYV challenge. Flow cytometric analysis was conducted to evaluate the impact of vaccination on the cellular immune response to wt MAYV infection of mice previously vaccinated with wt MAYV or MAYV/IRES. Six-week-old BALB/c mice were inoculated with 2 × 10^5^ PFU/50 μL of wt MAYV or MAYV/IRES via intraplantar (i.pl.) route and 28 days later mice were challenged with 2 × 10^5^ PFU/50 μL of wt MAYV at the same hind paw. After 3 days, animals were culled, and splenocytes were isolated for flow cytometry. Cells were incubated in the presence of the protein transport inhibitor brefeldin A, followed by staining with antibodies against surface markers expressed by DCs (**a–d**), monocytes/macrophages (**e–g**), T CD8^+^ (**e–i**) and T CD4^+^ (**h–l**) lymphocytes. Splenocytes were then fixed, permeabilized and incubated with antibodies against intracellular TNF-α (**c**,**g**), IL-17 (**k**) and Foxp3 (**l**). Gating strategy is illustrated in Supplemental Fig. 4. Data is expressed as mean ± SEM of at least 5 animals. MOCK- not-infected. *p < 0.05 when compared to control uninfected cells (MOCK). ^#^for p < 0.05 when compared to wt MAYV vaccinated mice, as assessed by one-way ANOVA followed by Newman-Keuls post-test.

The protection afforded by vaccination with MAYV/IRES was accompanied by a greater immune response in the spleen (Fig. 7a–l and Fig. S4). MAYV/IRES vaccinated mice showed higher accumulation and activation of splenic DCs (Fig. 7a–d), showing higher expression of the activation markers CD11b, CD86, as well increased TNF-α production. MAYV/IRES vaccinated mice also showed higher activation of splenic macrophages (Fig. 7e–g), as indicated by the higher expression of CD86 and TNF-α in comparison to wt MAYV vaccinated mice. Greater activation of T lymphocytes was also detected following MAYV/IRES immunization (Fig. 7h–l). Splenic CD8^+^ lymphocytes showed increased expression of the activation marker CD44 in MAYV/IRES versus wt MAYV vaccinated mice (Fig. 7h–i). Indeed, compared with wt MAYV vaccinated mice, MAYV/IRES induced IL-17A-producing T CD4^+^ lymphocytes in the spleen, with no change in the frequency of CD25^+^Foxp3^+^ Treg cells (Fig. 7k,l). Taken together, our results show that vaccination of immunocompetent mice with the live attenuated MAYV/IRES virus was safe and induced full protection against a virulent WT-MAYV strain challenge.

### Safety and immunogenicity of the vaccine in A129 deficient mice

**Figure 8:**
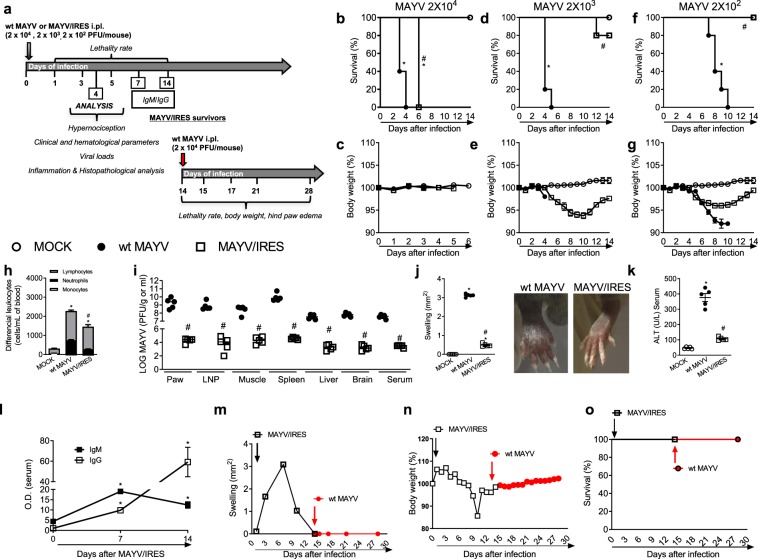
Immunogenicity and efficacy of the live attenuated MAYV/IRES vaccine in A129 mice. (**a**) Experimental design. (**b–g**) 8-week-old A129 mice were inoculated with 2 × 10^4^, 2 × 10^3^ or 2 × 10^2^ PFU/50 μL of either MAYV/IRES or wt MAYV strains and lethality rates (**b**,**d**,**f**) and body weight (**c**,**e**,**g**) analyzed every 12 hours until day 14 post-virus inoculation. Results are shown as % survival or body weight. Intermediary inoculum (2 × 10^3^ PFU/mouse) was chosen for further experiments (**h–o**). Analysis were conducted 4 d.p.i. h) Total and differential cell counts of inflammatory cells in the blood. (**i**) Plaque assay analysis of hind paw, PLN, quadriceps muscle, spleen, liver, brain and serum. Results are shown as the log of PFU per/g of tissue or PFU per/mL of serum. (**j**) Footpad swelling was assessed by measuring the height and width of the perimetatarsal area of the hind foot. Results are shown as mm^2^. (**k**) ALT levels in serum. Results are show as U/L of serum. (**l**) Anti-MAYV IgM and IgG titers in serum collected 0, 7 and 14 d.p.i. Results are expressed as O.D. (**m**) Footpad swelling was assessed by measuring the height and width of the perimetatarsal area of the hind foot before and after wt MAYV challenge. Results are shown as mm^2^. (**n**) % of body weight. (**o**) % of survival. Results were expressed as median (**i**) or mean ± SEM (**b–h** and **j–o**) and are representative of two experiments. *p < 0.05 when compared to control uninfected mice (MOCK). ^#^p < 0.05 when compared to naïve wt MAYV infected group, as assessed by two-way (**b–g**) or one-way ANOVA followed by Newman-Keuls post-test (**h–o**).

Type I interferons are key mediators in the control of arboviral infections^28^. A129^−/−^ mice lack functional type I interferon receptors and are therefore very susceptible to severe alphavirus infections^29^. They have been also used as a lethal model for alphavirus vaccine safety and challenge studies^25,29,30^. Here we evaluated the safety and efficacy of the live-attenuated MAYV/IRES vaccine in A129^−/−^ mice. Figure 8a shows the experimental scheme used.

Initial experiments evaluated the safety of the vaccine versus the WT strain in A129^−/−^ mice. Eight-week-old A129^−/−^ mice were inoculated with 2 × 10^4^ or 2 × 10^3^ or 2 × 10^2^ PFU/50 μL of either MAYV/IRES or wt MAYV strains and lethality rates and body weight analyzed every 24 hours until day 14 after inoculation. Results show that at the highest inoculum both wt MAYV and IRES-inoculated mice succumbed to infection; however, lethality was significantly delayed with the MAYV/IRES strain in comparison to the virulent WT strain (Fig. 8b). There was no weight loss in any of the groups as animals succumbed very quickly to infection (Fig. 8c).

Infection of mice with a 10-times lower inoculum (2 × 10^3^ PFU) resulted in death of all mice inoculated with the wt MAYV strain by day 5 (Fig. 8d). In contrast, 80% of animals inoculated with MAYV/IRES survived the inoculation (Fig. 8d). Weight loss was not observed in mice infected with the WT strain; these mice died by day 5. However, in mice inoculated MAYV/IRES, there was progressive body weight loss starting at day 6 and lasting until day 10 after inoculation. At day 14, the mean body mass from the MAYV/IRES inoculated group was similar to non-infected animals (Fig. 8e).

Finally, in A129^−/−^ mice inoculated with 2 × 10^2^ PFU of the wt MAYV strain induced progressive body weight loss (Fig. 8g), which culminated in 100% of death by day 10 after virus inoculation (Fig. 8f). In contrast, the MAYV/IRES-infected group exhibited transitory body weight loss and 100% of survival (Fig. 7f,g). These results clearly suggest that low inocula of MAYV/IRES are safe in mice that are immunodeficient. Further experiments evaluated the inflammatory response and viral loads after vaccine and the WT virus inoculation in A129^−/−^ mice. The intermediate inoculum of 2 × 10^3^ PFU was used and analysis were performed at day 4 after infection, before the death of A129^−/−^ mice infected with the WT strain.

Figure 8h–k shows that inoculation of 2 × 10^3^ PFU of wt MAYV in A129^−/−^ induced increased total and differential leucocyte counts, especially of neutrophils and lymphocytes in blood (Fig. 8h), recovery of elevated viral loads in several organs such as paw, popliteal lymph node, muscle, spleen, liver, brain and serum (Fig. 8i), massive hind paw swelling (Fig. 8j) and acute liver injury assessed indirectly by elevated ALT transaminase levels in serum (Fig. 8k). Therefore, inoculation of the same virus inoculum of the MAYV/IRES vaccine strain was associated with lower viral loads in tissues, decreased inflammation and systemic markers of disease (Fig. 8h–k).

### Efficacy of the vaccine in A129−/− deficient mice

Next experiments evaluated whether the vaccine would induce antibodies and protective immunity in A129^−/−^ mice. Anti-MAYV humoral immune responses were evaluated at days 7 and 14 after MAYV/IRES inoculation. As seen in Fig. 8d, all mice infected with the control strain were dead by day 5. Figure 8l shows elevated levels of anti-MAYV-specific IgM on days 7 and 14 after MAYV/IRES inoculation in comparison to naïve mice. A slight elevation of anti-MAYV IgG levels was observed on day 7 followed by a 10 times increase of titers at day 14 (Fig. 8l). Once the MAYV/IRES vaccine induced anti-MAYV specific antibodies, we challenged MAYV/IRES survivors with a lethal inoculum of wt MAYV strain (2 × 10^3^ PFU). Vaccinated mice were fully protected against the lethal challenge (Fig. 8m–o). We observed absence of hind paw edema (Fig. 8m), body weight loss (Fig. 8n) or mortality (Fig. 8o) upon wt MAYV inoculation in vaccinated mice.

## Discussion

MAYV is a neglected arboviral disease. There is a potential risk that MAYV may follow CHIKV to cause large urban epidemics^31–36^, especially since new lineages may arise through recombination^21^. This possibility underscores the need for new ways of preventing or treating MAYV infection, including the need for an approved vaccine. Knowledge about MAYV-induced immunopathology frequently derives from studies with other alphaviruses^22^. In the present study, we described a novel model of MAYV infection in adult immunocompetent mice, which emulates the major manifestations of MAYV infection in humans. The major findings of the present study can be summarized as follows: (a) MAYV inoculation into young adult immunocompetent mice induced persistent articular hypernociception, transient viral replication in target organs, systemic production of several proinflammatory cytokines and chemokines; and specific humoral IgM and IgG responses; (b) Inoculation of a live-attenuated MAYV vaccine candidate (MAYV/IRES) in adult BALB/c mice induced stronger cellular and neutralizing antibody responses; (c) MAYV/IRES vaccination of immunocompetent and A129^−/−^ mice resulted in protection from disease induced by the virulent wt MAYV strain.

The age of animals in murine models of alphavirus infections has been considered a determinant factor in the susceptibility and severity of the disease. Neonates and young mice are more susceptible to infection and have been used to emulate alphaviral diseases^37–41^. Of note, Figueiredo and colleagues (2019)^41^ have shown that MAYV replication, restriction and induction of muscular inflammation are dependent on age, type-I Interferon response, and adaptive immunity. Indeed, MAYV infection of young (25–21-day-old) SV129 and BALB/c mice successfully caused arthritis and myositis, common hallmarks of MF^40,41^. As for other arboviruses, such as Zika and Dengue^42,43^, resistance of adult mice to alphavirus infection is especially due to innate immune components such as type I interferons, which quickly provide an antiviral state of non-infected cells^28,29,41^. Accordingly, infection of type I interferon receptor-deficient mice (A129^−/−^) with CHIKV^29^, as well as infection of adult A129^−/−^ or recombination activation gene-1 deficient mice (RAG^−/−^) with MAYV^26,41^ provides a model that reproduces many of the clinical manifestations of human MF disease. Recently, MAYV infection of 3-4-week-old mice resulted in non-lethal models that emulated several clinical signs of MF in immunocompetent young-adult mice, such as footpad swelling, viremia and hepatic injury, although the arthritogenic effects of the virus were not investigated^26,44^. Adding to previous studies, in the present work MAYV infection of 5-6-week-old immunocompetent BALB/c mice caused arthritis and hypernociception, major manifestations of the disease in humans. Although infection of immunocompetent mice with MAYV did not reproduce symptoms to the same extent as in type I interferon receptor-deficient mice, it did induce most symptoms in the setting of a functional immune system, suggesting it may be a useful model for understanding the immunological response against MAYV. Indeed, using this model, we have demonstrated that the inoculation of a live-attenuated MAYV vaccine candidate (MAYV/IRES) in young BALB/c mice induced stronger cellular and specific humoral responses and protected from the clinical and laboratory changes induced by the WT infection.

Chronic joint inflammation and pain are the hallmarks of disease in patients with alphavirus infections^27,45^. Pain is one of the classical signs of the inflammatory process in which sensitization of the nociceptors is the common denominator^46^. The sensitization of primary afferent nociceptors leads to a state of hyperalgesia and/or allodynia, in humans, better described as hypernociception in animal models^47^. Articular hypernociception is usually indirectly evaluated in mice by mechanical and thermal tests^48^. Using these methods, we have shown significant hypernociception in response to mechanical stimulation upon Dengue virus infection^49^. Accordingly, we and others^50^ have demonstrated that infection of mice with wild type strains of MAYV induces increased mechanical hypernociception up to 21 days of virus inoculation. Interestingly, MAYV-IRES vaccination of mice was able to prevent the hypernociception induced by WT MAYV challenge.

Histopathological lesions, such as necrosis of cartilage and bone loss have been identified in experimental models of alphavirus infections such as CHIKV and RRV viruses. In patients with CHIKV, imaging studies have revealed the presence of tenosynovitis, synovial thickening, periostitis, periosteum proliferation and bone erosion. These clinical findings show that the acute disease may progress to chronic erosive arthritis^51,52^. Accordingly, we have demonstrated that MAYV infection also induces histopathological alterations in the ankle joints of mice, as characterized by loss of muscle and bone architecture. Tissues returned to basal conditions on day 21-post infection when hypernociception was not more evident. However, no evident histopathological alterations were detected in distal articulations such as knee joint, probably due to the age of mice and presence of active immune system in these mice, as compared to the other models in literature.

Evaluation of the profile of Inflammatory mediators in the spleen from wt MAYV infected mice revealed that MAYV induced a strong inflammatory response in the early stages of infection, characterized by production of several cytokines such as TNF-α, IL-6, INF-γ, IL-1β and IL-17 and well the chemokines CXCL-1, CCL2, CCL3, CCL4 and CCL5. These mediators have also been observed in other alphaviral models of RRV and CHIKV, and more recently in patients with Mayaro Fever^27,38,53^. As in other alphavirus infections, MF causes persistent joint pain and is often disabling^54^. Joint pain is consequence of the action of inflammatory mediators, such as IL-6, TNF-α and IL-1β, as well as tissue damage, which sensitize nociceptors^55,56^. Accordingly, in patients with MAYV these mediators are also elevated and were associated with presence of chronic arthralgia^27^.

In the present study we also performed an in-deep characterization of a live-attenuated vaccine (MAYV/IRES), which showed beneficial responses in infant immunocompetent CD1 mice and in Interferon-receptor deficient mice^25^. The live-attenuated MAYV/IRES strain did not replicate to comparable titers as the WT strain in adult BALB/c and A129^−/−^ mice; however, the vaccine candidate elicited a robust and specific antibody responses, similar to that observed with the wt MAYV strain. The vaccine analyzed in this work showed to confer protection against MAYV challenge and to be safe in an adult immunocompetent model. When compared to the WT, the MAYV/IRES strain induced lower levels of all the cytokines and chemokines analyzed showing lower inflammatory potential. *Ex vivo* analysis with splenocytes confirmed the ability of the MAYV/IRES vaccine to induce strong and long lasting cellular and humoral responses against wt MAYV strain. Accordingly, vaccinated mice challenged with wt MAYV showed reduced hypernociception, lower viral loads and decreased inflammatory markers in comparison to challenged non-vaccinated animals. MAYV/IRES vaccination also elicited augmented cellular responses, characterized by elevation of neutrophils and lymphocytes in the bloodstream. There was reduced level of MPO and increased NAG levels on the inoculated hind paw of vaccinated group as compared to wt MAYV-infected naïve mice. Plaque assay revealed a massive reduction of infectious virus recovery from the hind paw and a complete prevention of virus spread to the PLN, quadriceps muscle, spleen and serum. Indeed, histopathological analysis from the hind paw of MAYV/IRES-vaccinated mice revealed maintenance of tissue architecture, in contrast to the disruption caused by WT-MAYV injection. Finally, vaccinated mice showed lower levels of the cytokines IL-1β, IL-6, IFN-γ and TNF-α, as well as, the chemokines, CCL2, CCL3, CCL4 and CCL5. As these molecules are associated to monocytes response and to alphavirus normal response^37,38,57–59^, our data indicate lower level of monocyte activation by the vaccinal strain as compared to the WT virus. This is important for a vaccine for MAYV since activation of monocytes may contribute to the pathogenesis of MAYV-induced arthritis^53,59–63^. Finally, flow cytometric analyses of spleen leukocytes showed more robust immune response in MAYV/IRES immunized mice when compared to the WT strain. Immunization with MAYV/IRES induced splenic DC accumulation and activation, accompanied by a greater activation of splenic macrophages. MAYV/IRES also augmented T CD8^+^ immune response, as indicated by the higher frequency of cells TCD8^+^ cells expressing CD44. Indeed, we observed higher production of IL-17 by CD4^+^ Th17 cells in the spleens of in MAYV/IRES vaccinated mice. Taken together, our results show that vaccination of immunocompetent mice with the live attenuated MAYV/IRES virus was safe and induced fully protection against a virulent WT MAYV strain challenge.

We also tested this vaccine in a lethal model of MAYV infection in A129^−/−^ mice. In this model the vaccine maintains the behavior observed in the immunocompetent model with a major phenotype of protection. Of note, if given in a higher inoculum (2 × 10^4^), MAYV/IRES inoculation induced 100% lethality of A129^−/−^. Although this strain is attenuated, MAYV/IRES still replicates in host cells and A129^−/−^ are deficient in important innate immune components, more specifically the type I interferons α/β receptor. These molecules (IFN-α and β) play a significant role in preventing viral replication and protecting the host against arboviral infections^42,43,64–66^. Meanwhile, they are considered the gold standard models to evaluate virus replication and therapeutical strategies (drugs or vaccines) due their elevated susceptibility to infection. Those data are in accordance with a previous studies by Weise *et al*.^25^, and Webb *et al*.^26^. The vaccine proved to be safe, inducing good immunological responses with low viral loads and no local swelling. Since it seems only a matter of time for a large outbreak of MF to arise in Brazil, the development of a vaccine is crucial. Our data allow us to deduce that the vaccine candidate studied elicits good immunological response in the BALB/c model. The development of this vaccine must continue including testing additional animal models with a close attention to immune memory.

## Material and Methods

### Cells and viruses

The wild-type mayaro virus strain (wt MAYV) is a human isolate from Peru (in 2001) obtained from the World Reference Center for Emerging Viruses and Arboviruses at the University of Texas Medical Branch. The vaccine strain was developed from this isolate^25^. Viral replication and titration by plaque assay of both MAYV/IRES (vaccine) and wt MAYV strains was performed using the African green monkey kidney (Vero E6) cells obtained from the Banco de Células do Rio de Janeiro (BCRJ). Results are shown as the log of PFU per/g of tissue or PFU per/mL of serum. In some experiments, wt MAYV and MAYV/IRES were inactivated by heat (56 °C) for 60 min in the water bath. Inactivation was confirmed by plaque assay in Vero E6 cells.

### Ethical statement

This study was carried out in strict accordance with the ethical and animal experiments regulations of the Brazilian Government (Law 11794/2008). The experimental protocol was approved by the Committee on Animal Ethics of the Universidade Federal de Minas Gerais (CEUA/UFMG, Permit Protocol Number 160/2018) and Committee of Animal Experiments of the FAMERP (process #02078812.8.0000.5415). All surgeries were performed under ketamine/xylazine anesthesia, and all efforts were made to minimize animal suffering. Studies with MAYV were conducted under biosafety level 2 (BL2) containment at Immunopharmacology Laboratory from Instituto de Ciências Biológicas (ICB) at the Universidade Federal de Minas Gerais (UFMG). Briefly, male 6-week-old (18–20 g) immunocompetent BALB/c mice provided from Biotério Central at UFMG were used. In some experiments, 8-week old male A129^−/−^ SV129 strain of mice that are deficient to type I Interferon (α/β) receptors obtained from Biotério de Matrizes da Universidade de São Paulo (USP) were utilized. Mice were kept at 23 °C with a 12 h light/dark cycle and food and water *ad libitum*. Infection was performed by subcutaneous (s.c.) inoculation of 2 × 10^5^ PFU/50 μL/paw into the plantar surface of the right hind paw with of the wt MAYV group or vaccine (MAYV/IRES group) strains of MAYV. Groups of 5–6 mice were euthanized at 1, 3, 5, 7, 14 and 21 days post-infection (d.p.i.) for serum, spleen, popliteal lymph node, paw and joints analysis. Negative control group was inoculated with 50 μL of cell culture supernatant by s.c. intraplantar inoculation and euthanized at day 21.p.i.

MAYV/IRES vaccine immunization was performed as previously described by s.c. intraplantar inoculation of 2 × 10^5^ PFU/50 μL/paw followed by a challenge with 2 × 10^5^ wt MAYV strain at day 28 post-immunization. Analysis was performed at day 3 post-challenge.

### Clinical and hematological parameters

Lethality rates and body weight were analyzed every 24 hours. Disease signs (presence of ruffled fur, partial or complete hindlimb weakness or paralysis and loss in body weight) were monitored daily. Moribund mice with 20% or more body weight loss were euthanized. For hematological analysis, blood was obtained from the cava vein in heparin-containing syringes at the indicated times after infection. Total leucocytes count was obtained by using a Neubauer chamber. Leukocyte (lymphocytes, monocytes and neutrophils) differential counts were subsequently quantified microscopically from blood smears of each mouse.

### Hypernociception assessment

Hypernociception was assessed as described previously^49,67^. Briefly, mice were placed in acrylic cages with a wire grid floor 15–30 min before testing for environmental adaptation. To evaluate the articular hypernociception an electronic pressure-meter was used (INSIGTH Instruments, Ribeirão Preto, SP, Brazil)^48^. Mice were tested before (baseline) and 1, 3, 5, 7, 14 and 21 days after virus inoculation. Results are expressed as Δ withdrawal threshold (g) calculated by subtracting zero-time mean measurements from the time interval mean measurements.

### Plaque reduction neutralization test (PRNT)

PRNT_50_ was used to quantify the circulating levels of anti-MAYV neutralizing antibodies or to assess possible cross-reaction with CHIKV. Briefly, 24 well plates were seeded at a density of 4 × 10^4^ Vero cells/mL in each well. All serum samples were inactivated for 30 minutes at 56 °C before use. PRNT assays were performed as follows: serial dilutions of sera in a pool or from different days were mixed with constant quantities of wt MAYV (1 × 10^6^ PFU/mL) or with CHIKV strains (1 × 10^5^ PFU/mL) and then incubated for 1 hour at 37 °C. After incubation, the medium on each well was discarded and confluent cell monolayers were inoculated with 50 μL of the immunocomplex mixture. Then, the plate was incubated for another hour with 5% CO_2_ at 37 °C. After that, the virus-serum inoculum was discarded, and the cell monolayers were overlaid with 1 mL of 2% carboxymethylcellulose (CMC) diluted in MEM supplemented with 1% heat-inactivated FBS and antibiotics. Plates were then incubated for 4 days in 5% CO_2_ at 37 °C. CMC medium was discarded by inversion and cells were fixed in 0.2% formalin for 1 h at rt. Cell monolayers were then stained with crystal violet solution (1% w/v) for 20 min. Neutralizing antibody titers were expressed as the reciprocal of the highest initial serum dilution inhibiting at least 50% of plaque formation compared with the virus control titration.

Supplementary Table 1 shows that neutralizing antibodies were induced by MAYV/IRES inoculation, reaching satisfactory titers 5 days after inoculation. To determine if this response could cross-react with other alphaviruses, a pool of sera from five animals inoculated with MAYV/IRES strain and collected at each time point was tested against CHIKV (Sup. Table 2). PRNT_50_ values revealed that the MAYV/IRES vaccine is effective in inducing humoral immunity against MAYV but not CHIKV.

### Evaluation of cytokines and chemokine levels by ELISA

Cytokine and chemokine levels in samples were evaluated by ELISA using commercial kits according to manufacturer instructions (R&D Systems, Minneapolis, MN). Results were expressed as cytokine/chemokine picograms (mean ± error) normalized to 100 mg of tissue or 1 ml of serum.

### Macrophage and neutrophil recruitment

Macrophage and neutrophil recruitment in mice spleen and liver was indirectly determined by N-acetylglucosaminidase (NAG) or myeloperoxidase (MPO) enzyme activity evaluation, respectively, as previously described^68^.

### *Ex vivo* splenocytes stimulation

Splenocytes from all experimental groups (MOCK, wt MAYV and MAYV/IRES) were isolated at day 28 p.i. Briefly, spleens were macerated using a pestle and a mortar with RPMI medium containing antibiotics. Cells were then isolated, passed through a 0.70 μm filter and conditioned on ice. Cell solution was then centrifuged for 10 min at 300 × *g*. Then, pellet was incubated with lysis buffer ACK (0.15 M NH_4_Cl, 1 mM KHCO_3_, and 0.1 mM EDTA) for 5 min, it was added PBS to a final volume of 30 mL and the solution was centrifuged for 10 min at 300 × *g*. The cells were resuspended in RPMI (10% FBS, antibiotics). 5 × 10^5^ cells per well were then cultivated in 96 well plates. Splenocytes cultures were then stimulated with 5 × 10^5^ heat-inactivated wt MAYV and MAYV/IRES virus (MOI of 1). Some wells received a polyclonal stimulus - ConA at concentration of 2 μg/mL as positive control, or only RPMI media (negative control). For flow cytometry, unstimulated splenocytes were seeded in 96-well plate and incubated with brefeldin A (BD GolgiPlug) for 5 hours, at 37 °C, 5% CO_2_ before antibody staining. Cells were spun to remove the supernatant and surface stained with fluorescently conjugated antibodies anti-CD3, -CD4, -CD8, -CD19, -CD11c, -F4/80, -CD11b, -CD25 and -CD86 (Biolegend). Next, cells were fixed with 1% formaldehyde, permeabilized for 20 minutes with permeabilization buffer (BD, Perm/Wash Buffer) followed by incubation with antibodies against intracellular TNF-α, IL-17 and Foxp3. Cells were analyzed in a FACS Canto II cytometer. Gating strategy is illustrated in Supplemental Fig. 4.

### Histopathological analysis

After euthanasia, mice hind paws and knees were collected and fixed in 10% neutral buffered formalin (pH 7.2). Samples were processed for routine histology, sectioned (5 μm), and stained with Hematoxilyn and Eosin. The hind paws were evaluated using a semi quantitative histopathological score adapted from a previous study^69^. The maximum score grade was 7: inflammatory infiltrate [from 0 (absent) to 4 (severe)], and loss of muscle architecture [from 0 (absent) to 3 (intense)]. The knee score evaluated the inflammatory infiltrate [0 (absent) to 4 (severe)], synovial hyperplasia [0 (absent) to 3 (extensive)] and bone resorption [0 (absent) to 2 (extensive)], being the maximum score 9, following the criteria described previously^69^. All the analyses were carried out using a single-blinded model.

### Anti-MAYV IgM and IgG quantification

Antibody quantification in serum from MAYV-infected mice was performed by an indirect ELISA assay as adapted from Costa *et al*.^70^. Briefly, the same MAYV strain used in infections was UV-inactivated (60’) and diluted in 0.01 M carbonate buffer (pH 9.6) in a concentration of 1 × 10^6^ PFU per well of microtiter plates and incubated overnight. Plates were washed three times and blocked with bovine serum albumin 1% for 2 h and subsequently washed. Then, four dilutions of each serum sample from 1:50 to 1:6,250 were plated in duplicates and incubated for 3 h. After another wash step, plates were incubated for 2 h with peroxidase-conjugated anti-mouse IgG or IgM (Southern Biotech). Toward, ortho-phenylenediamine was used as a substrate, and the reaction was stopped with 1 M sulfuric acid. The absorbance was measured at 492 nm. Samples of MAYV-infected mice were considered positive in the first dilution in which mock samples were negative and were expressed as optical densities (O.D.).

### Statistical analysis

Results are shown as mean ± standard error (SEM), except viral loads that were expressed as median. Body weight is converted to a percentage and loss/weight gain calculated by subtracting the basal levels (obtained prior to infection) compared to control and infected mice. Differences were compared using analysis of variance (ANOVA) followed by Student–Newman–Keuls post hoc analysis. All analyzes were performed using the GraphPad PRISM software 6.0 (GraphPad Software, USA). Results with a p < 0.05 were considered significant.

## Supplementary information

Supplementary information

## Data Availability

Data generated and analyzed during this study are included in the published article (and its supplementary information files). Materials and data are also available from corresponding author on request.
