# Supplementary material for: In-depth characterization of a novel live-attenuated Mayaro virus vaccine candidate using an immunocompetent mouse model of Mayaro disease
Source: Sci Rep. 2020 Mar 24;10:5306. doi: 10.1038/s41598-020-62084-x (PMC7093544; doi:10.1038/s41598-020-62084-x)

SUPPLEMANTARY INFORMATION

In-depth characterization of a novel live-attenuated Mayaro virus vaccine candidate using an immunocompetent mouse model of Mayaro disease

Mânlio Tasso de Oliveira Mota[1#]; Vivian Vasconcelos Costa[2#*]; Michelle Amantéa Sugimoto[2]; Georgia de Freitas Guimarães[1], Celso Martins Queiroz-Junior[2]; Thaiane Pinto Moreira[2]; Carla Daiane de Sousa[2]; Franciele Martins Santos[2], Victoria Fulgêncio Queiroz[2]; Ingredy Passos[2]; Josy Hubner[2]; Danielle Gloria Souza[2]; Scott C. Weaver[3]; Mauro Martins Teixeira[2]; **Maurício Lacerda Nogueira[1*]**.

Supplementary Table 1. PRNT$_{50}$ in the sera of mice inoculated with MAYV/IRES strain

	**d.p.i.**				
Animal	**3**	**5**	**7**	**14**	**21**
**1**	1:20	1:320	1:40	1:320	>1:640
**2**	<1:20	>1:640	1:40	1:320	>1:640
**3**	<1:20	1:320	1:80	1:320	>1:640
**4**	<1:20	1:320	1:80	>1:640	>1:640
**5**	1:20	1:160	1:80	>1:640	>1:640

d.p.i. = days post-infection.

Supplementary Table 2. PRNT$_{50}$ in *pool* of MAYV/IRES inoculated mice sera

	**d.p.i.**				
	**3**	**5**	**7**	**14**	**21**
MAYV	>1:320	>1:320	1:40	>1:320	>1:320
CHIKV	1:160	1:160	1:160	1:160	1:160

Supplementary legends

Figure S1: Characterization of MAYV disease in immunocompetent mice. 6-week-old BALB/c mice were inoculated or not with MAYV (2×10^5 PFU/50 μL, i.pl.) and hind paw histopathological analyses was performed along the kinetic of infection. Figure S1A shows semi-quantitative analysis of hind paw sections of control and MAYV-infected mice, 1, 3, 7, 14 and 21 d.p.i. (Scale Bar - 100 μm). The images presented are representative of an animal on each day. Results were expressed as mean ± SEM and are representative of two experiments. * $p<0.05$ when compared to control uninfected mice (MOCK), as assessed by one-way ANOVA followed by Newman-Keuls post-test.

Figure S2: Serum cytokine levels in MAYV-infected immunocompetent mice. 6-week-old BALB/c mice were inoculated or not with MAYV (2×10^5 PFU/50 μL, i.pl.) and serum analyses of pro-inflammatory cytokines were performed along the kinetic of infection. Levels of: A) IL1-β, B) TNF-α and C) IL-6 in the serum of MOCK- and MAYV-infected mice. Results are expressed as pg/mL of serum. Data is representative of two experiments expressed as mean ± SEM. *$p<0.05$ when compared to control uninfected mice (MOCK), as assessed by Mann-Whitney test.

Figure S3: wt MAYV or MAYV/IRES injection is not associated with knee joint histopathological changes. 6-week-old BALB/c mice were inoculated MAYV (2×10^5 PFU/50 μL, i.pl.) and knee histopathological analysis was performed along the kinetic of infection. Figure S3A shows semi-quantitative analysis of knee joint sections of MOCK- and MAYV-infected mice, 1, 3, 7, 14 and 21 days after infection (Scale Bar - 100 μm). The images presented are representative of an animal on each day. Results were expressed as mean ± SEM and are representative of two experiments. * $p<0.05$ when compared to control uninfected mice (MOCK), as assessed by one-way ANOVA followed by Newman-Keuls post-test.

Figure S4: Gating strategy for accumulation and activation of leukocytes in the spleen wt MAYV and MAYV/IRES vaccinated mice. Splenocytes from wt MAYV or MAYV/IRES vaccinated mice were obtained and incubated with Brefeldin A for 5h prior to staining. After exclusion of debris, and duplets, splenocytes were gated into $CD11c^+$ (DC), $CD11c^-F4/80^+$ (monocytes/macrophages), $CD4^+$ (T CD4) and $CD4^-CD8^+$ (T CD8) populations. Each population was expressed as mean ± SEM percentage of single cells. Activation of splenic DCs and monocytes/macrophages were measured by the expression of CD86, CD11b, and intracellular TNF-α, expressed as mean fluorescence intensity (MFI) ± SEM. T CD4 lymphocytes were further gated to investigate the production of intracellular IL-17A (Th17) and the expression of the Treg markers CD25 and Foxp3. Th17 and Treg population were expressed as percentage of T $CD4^+$ cells ± SEM. T CD8 lymphocyte activation was determined by the expression of CD44, expressed as percentage of T CD8 cells positive for this marker.

Supplementary Figure 1

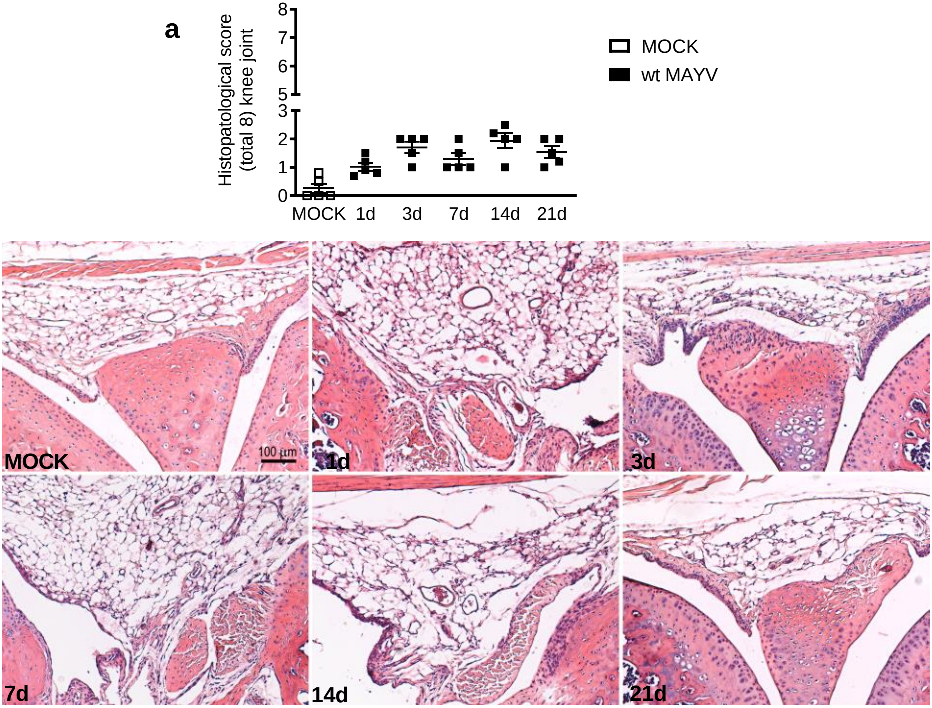

Supplementary Figure 2

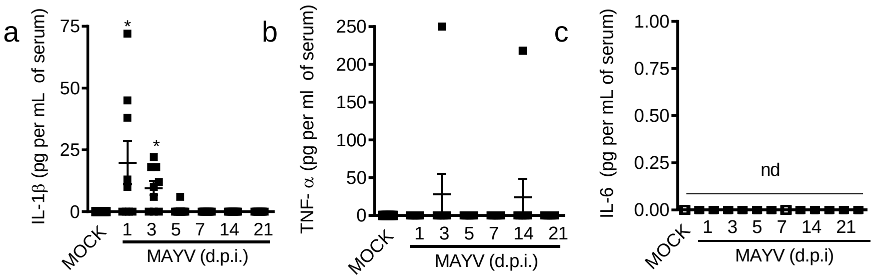

Supplementary Figure 3

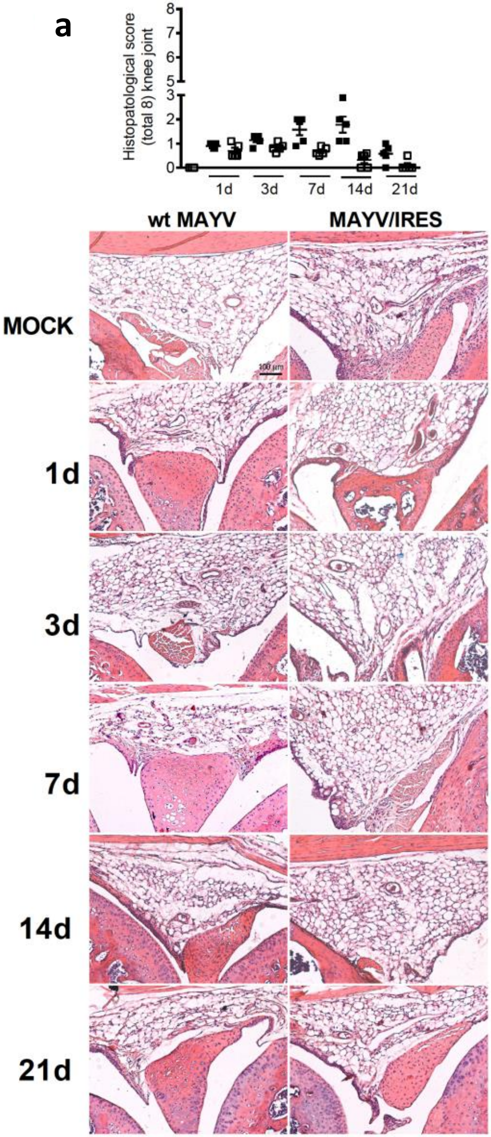

Supplementary Figure 4

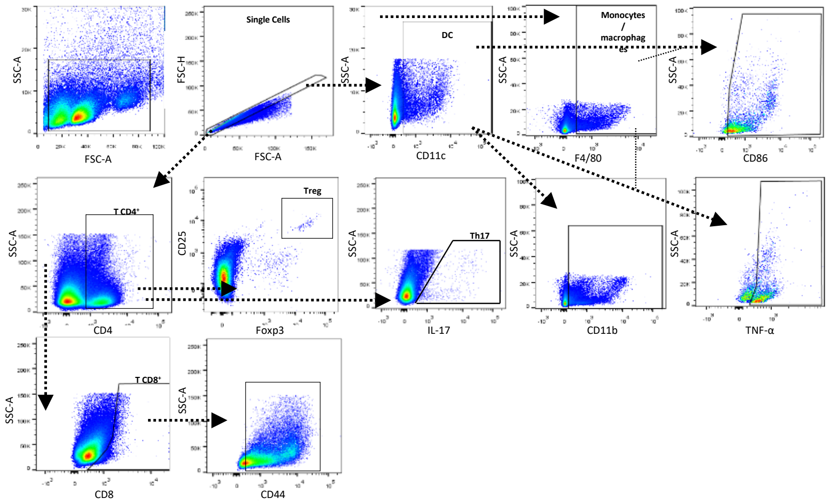

Supplement: Supplementary file 1 — Supplementary information [file 41598_2020_62084_MOESM1_ESM.pdf]
